# Evaluating the effectiveness of reasoning training in military and civilian chronic traumatic brain injury patients: study protocol

**DOI:** 10.1186/1745-6215-14-29

**Published:** 2013-01-30

**Authors:** Daniel C Krawczyk, Carlos Marquez de la Plata, Guido F Schauer, Asha K Vas, Molly Keebler, Stephanie Tuthill, Claire Gardner, Tiffani Jantz, Weikei Yu, Sandra B Chapman

**Affiliations:** 1 Center for BrainHealth®, The University of Texas at Dallas, 2200 Mockingbird Lane, Dallas, TX, 75235, USA; 2 Department of Psychiatry, University of Texas Southwestern Medical Center at Dallas, NE 210, 5323 Harry Hines Blvd., Dallas, TX, 75390, USA

**Keywords:** Cognitive rehabilitation, Traumatic brain injury, Neuroimaging, Neuropsychology, Reasoning, Memory, Attention

## Abstract

**Background:**

Individuals who sustain traumatic brain injuries (TBIs) often continue to experience significant impairment of cognitive functions mediated by the prefrontal cortex well into chronic stages of recovery. Traditional brain training programs that focus on improving specific skills fall short of addressing integrative functions that draw upon multiple higher-order processes critical for social and vocational integration. In the current study, we compare the effects of two short-term, intensive, group-based cognitive rehabilitation programs for individuals with chronic TBI. One program emphasizes learning about brain functions and influences on cognition, while the other program adopts a top-down approach to improve abstract reasoning abilities that are largely reliant on the prefrontal cortex. These treatment programs are evaluated in civilian and military veteran TBI populations.

**Methods/design:**

One hundred individuals are being enrolled in this double-blinded clinical trial (all measures and data analyses will be conducted by blinded raters and analysts). Each individual is randomly assigned to one of two treatment conditions, with each condition run in groups of five to seven individuals. The primary anticipated outcomes are improvement in abstract reasoning and everyday life functioning, measured through behavioral tasks and questionnaires, and attention modulation, as measured by functional neuroimaging. Secondary expected outcomes include improvements in the cognitive processes of working memory, attention, and inhibitory control.

**Discussion:**

Results of this trial will determine whether cognitive rehabilitation aimed at teaching TBI-relevant information about the brain and cognition versus training in TBI-affected thinking abilities (e.g., memory, attention, and executive functioning) can improve outcomes in chronic military and civilian TBI patient populations. It should shed light on the nature of improvements and the characteristics of patients most likely to benefit. This trial will also provide information about the sustainability of treatment-related improvements 3 months post-training.

**Trial registration:**

ClinicalTrials.gov Identifier: NCT01552473

## Background

The pervasiveness of traumatic brain injuries (TBIs) and the functional problems they entail are often tragic for injured individuals, their relations, and society as a whole. TBI is a major cause of death and disability, with as many as 1.7 million cases per year [1], and has a high incidence in adolescents and young adults [2]. TBI incidents in the US commonly occur because of vehicle accidents, falls, and assaults [2]. In the past decade, the incidence of TBI has risen sharply, to an estimated 19 percent in military populations, because of the wars in Iraq and Afghanistan [3,4].

Traumatic brain injury (TBI) survivors, including those with mild injuries, may continue to experience significant difficulties in functioning relative to optimal levels at work, home, or in the community even years post-injury [5,6]. Impairments are especially prominent in tasks and daily activities that draw upon higher-order cognitive processes mediated by the frontal lobes, an area of the brain that is exceptionally vulnerable to trauma. Rehabilitation efforts to remediate frontal lobe-related cognitive impairments are relatively rare, especially in chronic stages of recovery. Traditional cognitive training targets remediation of specific cognitive skills (e.g., memory) that do not necessarily draw upon frontal lobe-dominant integrative functions. Additionally, access to and availability of functionally relevant theoretical frameworks to guide brain training are often limited. Furthermore, TBI training studies rarely include imaging measures to characterize structural and functional brain changes.

To address prior shortcomings, researchers have begun developing cognitive rehabilitative therapies that target frontal lobe-mediated top-down modulatory processes. Top-down control processes are deliberate, effortful cognitive processes that both focus attention on task-relevant stimuli and screen out irrelevant distractions [7,8]. At the level of the brain, top-down modulation involves governing the operations of enhancement and suppression of neural activity based on the relevance of the information to task goals. Increasingly, neuroimaging results highlight the role of the prefrontal cortex in top-down modulatory tasks [9]. Accordingly, training frontal-mediated top-down processes in adults with TBI could be beneficial in restoring and improving higher-order cognitive functions.

In the current randomized control trial, we study the efficacy of a functionally relevant cognitive training program applied to individuals who are experiencing the effects of chronic TBI. The top-down training program labeled SMART (Strategic Memory Advanced Reasoning Training) adopts an integrative approach to train functionally relevant complex reasoning abilities (versus specific skills). This integrative approach focused on frontal lobe functions has shown promising results in a preliminary study [10]. SMART is compared to an equally engaging education-based program labeled BHW (Brain Health Workshop). Both SMART and BHW are short-term, intensive (18 h of training over 8 weeks) group training programs that are comparable with regard to training time, amount of information, group discussions, and homework assignments. The overall goal of this trial is to examine how training integrative frontal lobe-mediated processes might improve functioning in brain injury survivors, including military service and civilian populations. We include a range of individuals with different injury types and functional abilities. We use a broad variety of assessment tools, including cognitive, neuroimaging, and functional measures, to compare the training groups.

### Aims

Our overall goal is to improve the fidelity of TBI diagnoses and to achieve higher levels of functional recovery in soldiers and civilians who have suffered mild to moderate TBIs and are at the chronic stage of brain recovery. This study is also to determine the efficacy of an empirically and theoretically driven framework to enhance frontal lobe-mediated reasoning ability in individuals with TBI, given a relatively short training duration, on trained and untrained cognitive skills, on brain changes, and on measures of real-life function.

Toward these aims, this trial is enrolling both soldiers and civilians with a TBI (approximately 50 mild and 50 moderate chronic TBI patients). We use cognitive tests (assessing memory, reasoning, and comprehension abilities), functional MRI scans (performing tests of cognitive function while the subject is receiving an MRI scan), and white matter maps constructed using diffusion tensor imaging (DTI) scans. The MRI scans will be used to provide biomarkers of the contributions of different brain regions to performing cognitive tasks (e.g., memory, reasoning, etc.), as well as assessments of brain efficiency, functional brain connectivity, and brain morphology. We use these measurements to gain an understanding of each individual’s cognitive skills and neural measures prior to cognitive intervention. These measures also serve as indicators of the baseline function of each soldier or civilian, to be compared with after intervention, at which point they undergo post-training cognitive, MRI, and DTI assessments, enabling us to make outcome comparisons between the two different cognitive interventions. Finally, we conduct a follow-up assessment with neuropsychological and cognitive measures and neuroimaging 3 months after the interventions to assess how individuals maintain any functional changes that may occur because of the cognitive interventions.

We are targeting this intervention toward mild and moderate TBI participants, who have relatively high functioning skills. The demands of the training can be too high for some individuals falling into the more severe range, in the frequency, duration, and type of strategies and skills emphasized. We also aim to address the high level of need placed upon studies of milder TBI cases, particularly with military populations. This priority is also emphasized by the sponsoring agency, the US Department of Defense, advocating for more studies of mild TBI under the funding mechanism supporting this work.

**Aim 1.** Examine the short-term effects of SMART compared to BHW on cognition and real-life outcomes in soldiers and civilians with TBI.

#### Hypotheses related to Aim 1

A. Subjects who receive SMART show a greater increase from baseline on measures of attention, memory, and reasoning when compared to those who receive BHW.

B. Subjects who receive SMART show greater improvements in untrained cognitive measures that engage executive functions (e.g., measures of inhibition, non-verbal reasoning, task switching, working memory, and fluency) compared to those who receive BHW.

C. Subjects who receive SMART show greater improvement on rating scales of life skills functioning than those who receive BHW.

**Aim 2.** Examine changes in functional magnetic resonance imaging (fMRI) measures as a result of SMART versus BHW.

#### Hypotheses related to Aim 2

A. Subjects who receive SMART show greater modulation of ventral temporal regions previously shown to be a biomarker of attention toward relevant information relative to BHW participants.

B. Subjects who receive SMART will show greater activation of the frontal cortex relative to BHW participants following the intervention.

**Aim 3.** Determine whether the effects of SMART versus BHW training are maintained at a time point 3 months after cognitive intervention training.

#### Hypotheses related to Aim 3

A. The effects of SMART on abstract reasoning ability are maintained at 3 months after training, with no predicted change in the BHW group.

B. The effects of SMART on untrained executive function measures are maintained at 3 months after training, with no change in the BHW group.

C. The effects of SMART on daily life function measures are maintained at 3 months after training.

## Methods/design

### Design

This is a two-arm, randomized, double-blinded (with respect to scoring and data analysis), single-center, controlled clinical trial of patients with mild or moderate TBI with a 3-month follow-up phase. Outcome variables include cognitive, daily-life functioning, and brain-based MRI measures.

All subjects provide written informed consent prior to participating in any study procedures. Subjects are assigned to either of two training conditions (SMART, BHW) and are categorized by population (civilian, military) and severity of TBI. To make training groups less heterogeneous with respect to learning difficulties, participants are segregated into separate TBI groups (mild, moderate) for training purposes. The experiment is administered in four phases: pre-training, training, post-training, and delayed post-training. Each of the three testing phases involves taking a variety of measures at different times relative to the training phase.

In the pre-training phase, we gather assessment data from neuropsychological measures that tap into several different cognitive domains; take experimental measures that assess functions such as reasoning, memory, and comprehension; and include neuroimaging measures (task-based and resting fMRI, DTI, arterial spin labeling (ASL), and structural imaging). The training phase consists of two arms, each containing one of the cognitive interventions. The post-training phase consists of gathering data on versions of the same neuropsychological, cognitive, and neuroimaging measures previously collected at the pre-training phase. These measures are used to study the degree to which there are changes in cognitive and everyday life functioning after the training phase. It should be noted that in the current study we are using self-report questionnaire measures to determine improvements in everyday life. There have been recent reports in the rehabilitation literature indicating that ecological measures can also be useful and potentially more sensitive [11,12]. Given time limitations and the quantity of testing we have included in the current study, we have emphasized reasoning measures that incorporate aspects of real-world functioning, such as text comprehension (TOSL), and reasoning by analogy. Lastly, the delayed post-training phase consists of a final round of data collection on the neuropsychological, cognitive, and neuroimaging measures in order to assess the degree to which there is maintenance of improvements in cognitive and everyday life functioning and potentially to determine whether there are delayed improvements related to the training phase.

### Participants

A total of over 100 TBI patients will have been recruited for the study. We aim to enroll just over 50 civilians and 50 active or former military service persons. Participants are individually consented to participate in the entire study and participate in MRI scans. This study has been approved for research with human subjects by the Institutional Review Boards of the University of Texas Southwestern Medical Center at Dallas (IRB#8843) and The University of Texas at Dallas (IRB#11-43) and is in compliance with the Declaration of Helsinki.

#### Sample size justification

We performed a sample size analysis to determine the appropriate number of participants to enroll in order to assure that we would achieve adequate statistical power. This calculation was based on gist-reasoning effects from a prior pilot study conducted with TBI participants using similar cognitive interventions [10]. For an alpha level of 0.05, an anticipated effect size of 0.5 (medium), and a power of 0.8, we calculated a need for 102 participants (51 participants in each rehabilitation group) for a one-tailed (SMART greater than BHW) directional hypothesis. Additionally, we are actively recruiting military and civilian participants to include approximately equal numbers of each in the study. Likewise, we are recruiting to achieve a balance between mild and moderate TBI participants.

#### Estimate of feasibility

This is an ambitious study given the large sample size, the frequency of visits required by participants, and the duration of the visits. We believe that the number of participants and the extent of the tests and experimental procedures are both essential to providing the richness that we anticipate within this data set. Both performance sites, the Center for Brain Health and the Advanced Imaging Research Center, are centrally located in the Dallas-Fort Worth metropolitan area, allowing us access to a large and diverse population including numerous individuals who have sustained TBIs in either civilian life or military service. Over the past calendar year, we have been able to recruit an average of five individuals per month who qualify for the study. This level of patient flow indicates that we should be able to meet our sample size within 3 years, which is consistent with our duration of funding for the project.

#### Study inclusion criteria

Criteria for study inclusion are as follows:

• A Glasgow Outcome Scale Extended (GOS-E) [13] score between four and seven, inclusive.

• At least 6 months post-traumatic brain injury.

• Age between 19 and 65 years old (for the duration of the study).

• Ability to understand, read, and speak English.

• No current use of illicit drugs.

• No (self-reported) relevant pre-existing medical condition—including cerebral palsy, mental retardation, autism, controlled or uncontrolled epilepsy, pervasive developmental disorder, psychosis, or active behavioral disorder.

• Not currently pregnant.

Depending on participants’ behavior and ability to tolerate experimental procedures during the initial visit with clinical staff, we may also dismiss subjects who are judged, after discussion among the experimenters, to be unable to comply with or tolerate participation in the entire study. Participants who do not tolerate the MRI scanning protocol sufficiently (e.g., because of claustrophobia or difficulty keeping still) are removed only from subsequent scans. Our screening procedures and study design should preclude most such instances.

### Materials and apparatus

#### Screening materials

Participants are screened for inclusion and exclusion criteria during a structured screening interview, which includes the use of the GOS-E and a set of relevant demographic, medical, PTSD, and TBI questionnaires. PTSD is screened for in the current study, but the presence of PTSD symptoms is not exclusionary. While PTSD can influence performance in neuropsychological and experimental measures, we determined it would not be possible to study TBI effectively and exclude individuals with PTSD based on recent estimated comorbidity, particularly in military service-related TBI cases [14]. In addition to the severity level indicated by the GOS-E, in all cases we seek additional confirmatory evidence based on the Glasgow Coma Scale (GCS) and documentation of prior medical evaluations. Given that these secondary indicators are not always available, particularly for mild TBI participants, we rely on the functional level for defining the TBI severity in this study.

#### Outcome measures

Neuropsychological and functional outcome measures are used to assess changes associated with treatment in the following domains: gist reasoning, long-term memory, working memory, inhibitory control, verbal fluency, nonverbal reasoning, speed of processing, switching, attention, and relational reasoning.

The majority of measures are administered at the pre-training phase, post-training phase, and the delayed post-training phase. Each of the following measures relevant to initial entry into the study is administered to each subject only during the pre-training phase: a demographic log, data intake form, medical assessment form, post-traumatic stress disorder screening, first impression sheet, PCL-S screening for TBI exposure [15], verbal problem solving, WTAR [16], AUDIT [17], and WHO-ASSIST V3.0 [18].

All remaining tests are administered to all subjects at each of the three testing phases (pre-training, post-training, and delayed post-training). These tests include the SMART rating scale questionnaire, FSE [19], GOS-E [13] (the initial GOS-E score is used to assess severity; subsequent administrations of this scale serve as outcome measures relative to the initial administration), test of strategic learning (TOSL) (S. Chapman J. Hart, H. Levin, L. Cook, J. Gamino, unpublished data, 2009), selected subtests from the WASI [20] (vocabulary, matrix reasoning, and similarities), digit span from the WAIS-III [21], logical memory subtest from Wechsler Memory Scale-IV [22], selected subtests from the Delis-Kaplan Executive Function System (trail making test, color-word interference test, verbal fluency test, card sorting test), Digit Vigilance Test [23], Community Integration Questionnaire [24], listening span test [25], Hayling sentence completion test [26], BDI-II [27], Satisfaction with Life Scale [28], and the Awareness Questionnaire [29].

The VSLT (adapted from [30]) and picture analogies task (adapted from [31]) stimuli are presented on laptop computers (1,280/1,366 × 768 pixel screen). Alternative forms of neurocognitive tests are used when available to reduce practice effects. In evaluating all outcome data, we will correct for multiple comparisons within each testing subdomain (e.g., working memory, inhibitory control, etc.).

#### FMRI task materials

Three functionally identical versions (differing only in specific picture stimuli) of the fMRI face/scene selection task are being used [9].

#### Training materials

The materials for both training conditions are presented primarily in the form of slide show presentations, video clips, and written materials.

##### Strategic memory advanced reasoning training

The Strategic Memory Advanced Reasoning Training (SMART) utilizes a strategy-based approach to train individuals in abstract thinking ability [10,32]. Specifically, participants are trained in cognitive control strategies of *strategic attention, integration,* and *innovation*, which facilitate abstraction abilities [33]. Strategic attention involves blocking less relevant details to focus on important information. Integration incorporates strategies to abstract and create meanings or goals from information or tasks. Innovation focuses on generating and discovering novel concepts, ideas, and diverse goals and perspectives. The strategy instruction is hierarchical, with each strategy dynamically building upon previous strategies. The SMART program incorporates a wide range of discourse and task materials relevant in daily life contexts, such as planning an event, going on a job interview, learning from a lecture, or explaining a concept.

##### Brain health workshop

The BHW program focuses on teaching facts about brain functions and influences on cognition. This program was originally developed at the Rotman Institute, Canada [34]. The BHW includes topics such as an overview of brain anatomy, neuroplasticity, memory, attention and executive functions, aging and the brain, sleep and stress, diet and physical exercise, and social bonds and the brain. We adapted the curriculum to approximate the general structure of SMART training, including matching for the number of sessions, duration, discussions, and homework assignments.

### Procedure

As this study centers on contrasting two group-training programs, we form each participant cohort (one SMART and one BHW group) when sufficient numbers of participants have been enrolled. To balance assignment to conditions for both civilians and military service persons, we attempt to form cohorts with even numbers of each. When a cohort is formed, half of military service persons are randomly assigned to each training group, and half of civilians are likewise randomly assigned to each group.

**Figure 1:**
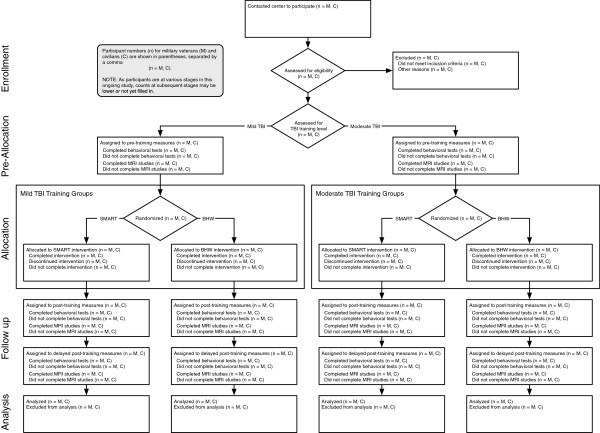
**CONSORT diagram.** This flow chart lists each phase of the study that is experienced by the participants. The chart also contains summary information about the procedures followed by the experimenters in implementing the study.

For each cohort, the entire study involves four phases: pre-training, training, post-training, and delayed post-training. Participants generally complete all four phases in about 24 weeks, or under 6 months. Each participant is individually tracked for assignment to conditions and participation in each phase of the study. Figure 1 depicts an overview of participant flow from initial contact through data analysis.

Each of the pre-, post-, and delayed post-training phases includes neuropsychological and cognitive testing (collectively, “testing”), as well as fMRI experimentation and structural MRI scanning (collectively, “imaging”). (Each of the three phases is a “testing-imaging” phase.) Table 1 shows the session type, timing, and duration for each study component at each phase. The training phase begins as soon as a cohort can be formed, usually within 3 weeks of the start of the pre-training phase. Training involves 12 sessions, with the first 10 sessions occurring in weeks 1–5 (2 per week), 1 session occurring in week 6, and 1 session taking place in week 8. The post-training phase is completed within 2 weeks of the end of training. The delayed post-training phase takes place 3 months after training.

**Table 1:** Study phase, session type, timing, and duration

**Phase**	**Type**	**Timing**	**Duration**
Pre-training	Testing, part 1	Within 3 weeks before session 1	1.5 h
	Testing, part 2	(Same as above)	2.5 h
	Imaging	(Same as above)	2 h
Training	Sessions 1–10	Weeks 1–5 (2 per week)	1.5 h
	Session 11	Week 6	1.5 h
	Session 12	Week 8	1.5 h
Post-training	Testing	Within 2 weeks after session 12	2 h
	Imaging	(Same as above)	2 h
Delayed post-training	Testing	At 3 months after session 12	2 h
	Imaging	(Same as above)	2 h

Pre-training testing occurs over two sessions lasting 4 (1.5 and 2.5) h. Data collected include both measures taken only at the pre-training phase and measures taken at all three testing-imaging phases. Each post- and delayed-post training testing session lasts approximately 2 h.

#### Neuropsychological and cognitive testing

During testing phases, neuropsychological and cognitive tests are administered. Tests with more than one variant are administered as follows: Those with two variants are administered in alternating fashion, with the first variant at the pre-training and delayed post-training phases and the second variant administered at the post-training phase. Those with more variants are administered in one of the six previously mentioned counterbalance orders or versions.

#### Test of Strategic Learning (TOSL)

The TOSL evaluates the ability to extract broad themes and targeted focal details from a passage of text, and it is a task based on a prior study of discourse comprehension (S. Chapman J. Hart, H. Levin, L. Cook, J. Gamino, unpublished data, 2009). Participants are presented with a single passage of text describing an individual and the details of their life, including several major themes that can be surmised from the passage overall. After reading the text passage or having it read aloud to them, the participant is asked to generate a condensed version of the story summarizing the deeper meanings that have been abstracted from the text followed by any lessons that could be learned from the story. The participant is next prompted by an experimenter to indicate whether specific details (e.g., the occupation of the individual described in the story) were present or not. Lastly, the participant is asked to generate details that they recall about specific details from the story. The TOSL is scored for measures of coherence, abstraction, memory, recognition, and accuracy for story content.

#### Visual Selective Learning Task (VSLT)

The VSLT was adapted from [30]. For our study, we use nine of their 16-word lists and present them visually, by computer screen, instead of aurally. Since the task is administered during each of the three neuropsychological testing phases, the experimental lists were divided into three sets (A, B, and C) of three lists.

Each list was formed as follows: Half of the words in each list were already in uppercase (U), and half were in lowercase (L). Within each list, we separately randomized U and L words. U and L words were then mixed so that each list had exactly one run of two and one run of three same-case words of each case. Runs of three were always at least one U and one L word (or run of words) away from list beginnings and endings. Runs of two were always at least one U and one L word (or run of words) away from same-case runs of three. Individual pseudorandom lists were then selected for sequence variety within sets.

At each study phase, participants are presented three word lists. Before each list, words are given point values (1 or 10 points) for recall based on their case (e.g., “UPPERCASE = 1 point, lowercase = 10 points,” or vice versa). After each list has been presented, participants are tasked with immediately trying to recall as many words from that list as possible, especially those of higher value.

In the ULU point-value scheme, first-list U words, second-list L words, and then third-list U words are high-value (10-point) items. In the LUL point-value scheme, the values of U and L words are reversed. At each study phase, participants receive a new list set (A, B, or C), but each set is presented with the same point-value scheme (either ULU or LUL).

#### Picture analogies task

The picture analogies task was adapted from [31]. Images and analogies used in the original set were upgraded and appended. Each is in the form A : B :: C : ? (i.e., A is to B as C is to what?), with four choice options for completing the analogy. For the current study, we use 3 practice and 42 experimental problems. Each problem comes in two versions—one with perceptual and semantic distractors among the options and one with no distractors.

Since the task is administered during each of the three neuropsychological testing phases, the experimental problems were divided into three sets of 14. At each phase, all 14 problems for that phase are presented in randomized order and then presented again in the same order. At first presentation, seven are presented with distractor options, and seven are presented with no distractor options. At second presentation, those problems previously presented with distractors are presented without distractors, and vice versa.

#### Neuroimaging

Each participant is transported a short distance by the experimenter to the Advanced Imaging Research Center at the University of Texas Southwestern Medical Center, where the MRI scanner is housed. After completing the University of Texas Southwestern MRI safety screening form, subjects view the experimental task pre-instructions under the guidance of the experimenter. Subjects are then prepared for and positioned in the scanner, and then the experimenter and a technician run the experiment and scanning protocols, respectively.

##### Functional MRI design

At imaging sessions, the fMRI Face/Scene Selection Task [9] is performed. The experiment consists of a total of 20 intermixed blocks divided into five runs. Among the four blocks in each run, three blocks are 1-back tasks, where the participant is instructed to look for a match of faces only or scenes only or both. One block is a categorization task, where the participant presses the right button for a scene and the left button for a face. Subjects need to view and recognize the category of the image in all conditions. However, in FACE, SCENE, and BOTH conditions, subjects have to decide whether the current image is a match to the previously viewed image of the relevant category, indicating matches with a right button press and non-matches with a left button press. Each image is presented for 590 ms, immediately followed by a fixation cross for a jittered inter-trial interval of 3, 5, or 7 s.

Prior to the above experimental task, an independent task is used to locate the regions selective for visual object categories, including face-selective [35] and scene-selective [36,37] areas. The functional localizer consists of seven 16-s blocks of properly displayed and jumbled grayscale faces, objects, and scenes, or a fixation cross. In order to ensure that participants are attentive during the localizer task, they are instructed to make simultaneous left and right button presses if they see an image repeat. This localizer task has previously been shown to reliably activate scene- and face-selective regions of the inferior temporal cortex [38].

Lastly, an additional 6 min of echo-planar imaging is acquired with participants in a resting state, for which they are instructed to lie still and remain awake in the scanner.

##### FMRI data acquisition

Imaging is performed on a 3-Tesla Scanner (Philips MR systems, Achieva Release 2.5.3.0). Functional images are acquired with an echo-planar image sequence sensitive to BOLD contrast (TE 30 ms, TR 2 s, α flip angle 70°). The volume covers the whole brain with a 64 × 64 matrix and 36 transverse slices (4 mm thickness with a 0-mm inter-slice gap) (voxel size 3.44 × 3.44 × 4 mm). Five runs consisting of 228 volumes of the selective attention task and one run of 168 volumes of the localizer task are acquired during the experiment. Structural images of individual subjects are acquired to serve as template images onto which the functional data will be mapped. The structural scans include a T1-weighted spin-echo image sequence with 36 transverse slices and a magnetization-prepared, rapid access gradient-echo image sequence with 160 sagittal slices.

##### Diffusion tensor imaging design—image acquisition

DTI images are obtained using a single-shot, spin-echo, echo-planar imaging sequence with field of view (FOV) = 240 mm, slice thickness/gap = 3/0 mm, approximately 45 slices, repetition time = 12,000 ms, echo time = 75.5 ms, flip angle = 90 degrees, number of excitations (NEX) = 2, and a matrix of 128 × 128. The diffusion-sensitizing gradients are applied at a b value of 1,000 s/mm^2^ per axis with 19 non-colinear directions and 3 b_0_ images. The acquisition time is 9 min. The voxel size is 2 × 2 × 3 mm interpolated (scanner default) to 1 × 1 × 3 mm. Three-dimensional (3D) T1-weighted structural FSPGR images are obtained with FOV = 240 mm, slice thickness/gap = 1.3/0 mm, approximately 130 slices, echo time = 2.4 ms, TR = 8 ms, flip angle = 25 degrees, NEX = 2, a matrix of 256 × 92, and an acquisition time of 6 min.

### Testing-imaging counterbalancing procedures

Each of four testing components, the TOSL, VSLT, picture analogies task, and fMRI study, is administered in one of six counterbalanced versions.

#### TOSL

As only one of the three TOSL versions (A, B, or C) is administered at each of the three testing phases, each participant receives one of six TOSL test-order permutations.

#### VSLT

The VSLT uses three list sets (A, B, and C) with two different letter-case point-value schemes (ULU, LUL; see below). Accordingly, each participant is presented the list sets in one of three orders under either of the two point-value schemes—that is, in one of six different ways.

#### Picture analogies task

Like the VSLT, the picture analogies task has three problem sets and two problem formats, which were used to generate six counterbalance versions.

Three problem sets are presented at different phases of the study (pre, post, and delayed post). At each study phase, problems are presented twice. On first presentation, half of the problems are presented with distractors (D) in their solution options, while the other half is presented with no distractors (N). On second presentation, problems previously presented with D options are presented with N options, and vice versa. The patterns DN-ND and ND-DN differ only in which half of problems are presented first with D or N options.

#### FMRI selective attention task

Finally, the fMRI selective attention task has three image sets to be presented in all six possible orders.

### Assignments to counterbalanced testing-imaging components

New participants are assigned to counterbalanced testing and imaging components by fixed rotation, except when assignments are unbalanced by discontinued participants. In such cases, any underrepresented counterbalance category is filled first.

### Testing and imaging

Each of the testing and neuroimaging sessions occurring at the pre-training phase, post-training phase, and delayed post-training phase is administered by testers/experimenters trained and experienced in their respective procedures.

### Training

Each participant is enrolled in either of two 12-session training programs. Both programs are run by a single instructor and two training facilitators. Both include group lectures, group discussions, individual homework assignments, and brief quizzes. The two programs are similar in several substantial ways, except with respect to the principles of interest for the study. For both programs, the instructors are available by phone or email to address any questions and concerns that participants may have. The intervention specialists conduct both SMART and BHW treatment groups to minimize any treatment bias that could be introduced by the presence of a specific intervention specialist. We also record the particular trainer who ran a given session and will statistically evaluate whether there is a significant effect of trainer evident in the data. Additionally, we record the particular training cohort group that an individual participated in and will evaluate whether there are significant effects of participation in specific groups that may influence the data in a biased way.

#### Random assignment to groups

To facilitate the training of each participant, groups are made more homogeneous by segregating individuals by their GOS-E scores. Subjects with scores of four and five are assigned to moderate TBI groups; those with scores of six or seven are assigned to mild TBI groups.

When a cohort of 12 participants with even numbers of civilians/military persons is formed (usually 6 of each), the cohort is divided into two groups of 6 members as follows: Half of the civilians and half of the military persons are randomly assigned to each group. Occasionally, after the formation of cohorts and groups, if it is determined that a participant cannot participate, that person is replaced by another individual. When a replacement must be made for one of the participants, the new participant is of the same TBI severity level and, preferably but not necessarily, from the same civilian/military population as the person removed.

Participants remain naïve regarding the particular content of their treatment group relative to that of the other treatment group. Additionally, we attempt to procedurally separate treatment groups as much as possible by holding the two types of group sessions at different times of day. Testing is performed individually by testers blind to the treatment group. We also encourage participants not to discuss the details of their group training experience with clinicians, testers, or others who may be involved in any phase of the study.

#### SMART

SMART consists of two overarching phases: serial, progressive teaching of the three sets of cognitive control strategies in the first few sessions and integrative training in the use of these strategies in increasingly complex classroom exercises and real-world situations in the remaining sessions.

#### Brain health workshop

BHW sessions involve the teaching of the several topics in serial order, along with exercises and games to reinforce the memorization of facts. Importantly, BHW does not involve learning cognitive strategies.

### Procedures for double blinding

Each experimenter who administers pre-testing, post-testing, delayed post-testing, and neuroimaging measures is kept blind to the treatment group of each participant. Further, the participants are not informed about the particular content of their treatment group relative to the other group in order to maximize the likelihood that all participants will participate as actively as possible within their particular assigned group.

## Discussion

This trial investigates training to improve cognition in individuals with mild and moderate chronic TBI. We are evaluating two treatment methods, SMART and BHW. While SMART is considered to be the active treatment arm, emphasizing cognitive strategies, the BHW provides information about the brain and, like SMART, in a clinician-led program with a group treatment experience. The efficacy of these treatments will be evaluated using experimental measures of higher-order reasoning and neuropsychological measures emphasizing working memory, memory for details, inhibitory control, verbal fluency, nonverbal reasoning, and cognitive switching, as well as neuroimaging measures, including fMRI, DTI, and functional connectivity MRI. The groups are measured prior to training, immediately post-training, and 3 months post-training.

One of the potential challenges that we anticipate during this trial is that we will likely have a fairly heterogeneous sample of TBI patients, which could make within-group measures more variable and thus reduce the statistical power of tests. This would be due to the fact that TBI varies both in severity and regions affected, depending upon the specific cause of the injury. In addition, the military population (and some civilians) may have been exposed to multiple concussions or traumatic incidents that complicate isolating causative events in a TBI diagnosis. The fact that we are recruiting from both civilian and military populations is likely to lead to further patient heterogeneity. From previous studies there is evidence that military-related TBI cases are different from civilian TBI cases [39,40].

Another important issue that may present a challenge to the completion of the study is the possible attrition of participants due to the frequency and number of sessions involved. This is particularly evident in the training portion of the study, as there are 12 sessions over a period of 8 weeks of attendance required. Additionally, we require a follow-up testing session 3 months after the completion of training and post-training testing-imaging. Given that participants may relocate or change contact information, it may be challenging to complete all study phases. In response to these concerns, we emphasize to participants the degree of commitment involved in the study during the initial recruitment visit and screening. Further, we keep contact on a monthly basis with each participant after they finish the training.

In summary, we are conducting this trial in order to better characterize and understand methods to treat chronic mild and moderate TBI. We aim to better understand how TBI patients respond to cognitive training and evaluate two particular forms of training. The information gathered from this study will potentially validate intensive and short-term cognitive interventions relevant for rehabilitation of individuals with mild to moderate TBI. If we are able to demonstrate that SMART is an effective treatment method that impacts cognition and everyday life functioning, this will be potentially useful for wider distribution and may help to improve work and life outcomes for individuals suffering from chronic TBI.

## Trial status

At the time of the submission of this manuscript, enrollment was ongoing.

## Abbreviations

ASL: Arterial spin labeling; AUDIT: Alcohol Use Disorders Identification Test; BDI®-II: Beck Depression Inventory-Second Edition; D-KEFS: Delis-Kaplan Executive Function System™; DVT: Digit Vigilance Test; FSE: Functional Status Examination; fcMRI: Functional connectivity MRI; GOS-E: Glasgow Outcome Scale-Extended; MRI: Magnetic resonance imaging; OSU: Ohio State University; SMART: Strategic Memory Advanced Reasoning Training®; TOSL: Test of Strategic Learning; TBI: Traumatic brain injury; VSLT: Visual Selective Learning Task; WAIS-III: Wechsler Adult Intelligence Scale-Third Edition; WASI: Wechsler Abbreviated Scale of Intelligence™; WHO-ASSIST V3.0: World Health Organization-Alcohol Smoking, and Substance Involvement Screening Test; WMS-IV: Wechsler Memory Scale-Fourth Edition; WTAR: Wechsler® Test of Adult Reading™.

## Competing interests

Due to prior evidence of the efficacy of the SMART program, The University of Texas at Dallas Center for BrainHealth is actively engaged in providing SMART to a variety of groups, including in the enterprise. The Center has also applied for a process patent for SMART.

## Authors’ contributions

DCK initiated the study, designed the study, wrote protocol drafts, and implemented the study. CM designed the study and wrote protocol drafts. GFS designed the study and wrote protocol drafts. AKV designed and implemented the study. MK designed and implemented the study. ST designed and implemented the study. CG designed and implemented the study. TJ designed and implemented the study. WY designed and implemented the study. SBC initiated the study, designed the study, wrote protocol drafts, and implemented the study. All authors contributed to and edited the protocol draft. All authors read and approved the final manuscript.
